# Medical News and Miscellany

**Published:** 1898-10

**Authors:** 


### Medical News and Miscellany.

**Dr. J. R. McCown** has removed from Willow City to Carl, Texas.

---

**The** address of Dr. Oliver O'Bar is changed from Galveston to Warrenton, Texas.

---

**The Austin District Medical Society** held its forty-fourth quarterly session in Austin on 29th Sept., ult., with a large attendance, and a most interesting program.

---

**Dr. C. T. Cooper** has removed from Ballinger, Texas, to San Angelo. Dr. Cooper, in company with Drs. Marberry and Tucker, will establish an infirmary at the latter place.

---

**Want** to buy a home in a railroad town. A good practice will be an inducement. A partnership and taking property later preferred. Address, Dr. E. P. Edwards, Jennings, Lamar County, Texas.

---

**Prof. H. P. Cook, M. D.,** of the faculty of the Medical Department, University of Texas, has been elected dean, vice Prof. A. J. Smith, M. D., who had served as dean temporarily by request, upon resignation of the deanship by Prof. J. F. Y. Paine, M. D.

---

**At the** September meeting of the Medical Department of the University of Nashville, the faculty unanimously decided that all students matriculating after the present session of 1898-'99, shall be required to attend four full courses of lectures, before being permitted to apply for graduation.

**Dr. T. J. Bennett,** a distinguished surgeon and physician of Austin, Texas, and one of the editors of the *Texas Medical News*, will leave for New York on the 10th inst., where he will spend some months at the polyclinics and great hospitals. After which he will visit the medical centers of Europe—putting on the finishing touches.

---

**Married.**—At Rockdale Texas, September 21, 1898, at the residence of the bride's parents, Miss Mary Sammie Osborne, daughter of Dr. William Osborne, to Mr. David C. Ellison, all of Rockdale. The JOURNAL acknowledges the courtesy of an invitation, and extends congratulations to the young people. May they "live long and prosper."

---

**It is not** surprising that the educated physicians will insist on saying "iodide of potash," and "chlorate of potash,"—instead of "iodide potassum" and "chlorate of potassa?" "Scarlatina" is another barbarism in general use, an English word with a Latin termination. It is only excelled in outrageousness by the use of —"uricacidæmia." Great scott! The fellow who first perpetrated that ought to be "kicked to death by a jassack" (but I wouldn't "like to be the one to do it.")

---

**The Austin Academy of Medicine and Surgery** was organized in Austin, Texas, last month. It is a successor to the Travis County Medical Society, which for twenty years has been in successful operation. The old society was dissolved by vote and the academy was organized. Dr. A. N. Denton, of Austin, the able and well-renown alienist, long time Superintendent of the State Insane Asylum was elected president, and Dr. C. O. Weller, of Austin, vice-president.

---

**The Louisiana Morphine and Cocaine Law.**—The legislature of Louisiana has passed a law making it unlawful to sell at retail any cocaine, morphine or opium, or its preparations, except under the written prescription of a practicing physician, such prescriptions not to be refilled. Violation of the law is punished by a fine of not over $100 or imprisonment for not over thirty days, or both, at discretion of the court.—*Texas Courier-Record.*

